# Outdoor Office Work – An Interactive Research Project Showing the Way Out

**DOI:** 10.3389/fpsyg.2021.636091

**Published:** 2021-04-12

**Authors:** Charlotte Petersson Troije, Ebba Lisberg Jensen, Cecilia Stenfors, Christina Bodin Danielsson, Eva Hoff, Fredrika Mårtensson, Susanna Toivanen

**Affiliations:** ^1^Department of Urban Studies, Malmö University, Malmö, Sweden; ^2^School of Health, Care and Social Welfare, Division of Sociology, Mälardalen University, Västerås, Sweden; ^3^Department of Psychology, Stockholm University, Stockholm, Sweden; ^4^Aging Research Center, Karolinska Institute, Solna, Sweden; ^5^School of Architecture, Royal Institute of Technology, Stockholm, Sweden; ^6^Department of Psychology, Lund University, Lund, Sweden; ^7^Department of People and Society, Swedish University of Agricultural Sciences, Alnarp, Sweden

**Keywords:** outdoor office work, sustainable working life, interactive research, work norms, human nature interactions, urban greenspaces

## Abstract

The physical boundaries of office work have become increasingly flexible. Work is conducted at multiple locations outside the office, such as at clients’ premises, at home, in cafés, or when traveling. However, the boundary between indoor and outdoor environment seems to be strong and normative regarding how office work is performed. The aim of this study was to explore how office work may be conducted outdoors, understanding how it is being experienced by office employees and identifying its contextual preconditions. Based on a two-year interactive research project, the study was conducted together with a Swedish municipality. Fifty-eight participants engaged in the collaborative learning process, including 40 half-day workshops and reflective group discussions, co-interviews, and participants’ independent experimentation of bringing work activities outdoors. Data was collected via interviews, group discussions and a custom-made mobile application. The results showed that a wide range of work activities could be done outdoors, both individually and in collaboration with others. Outdoor work activities were associated with many positive experiences by contributing to a sense of well-being, recovery, autonomy, enhanced cognition, better communication, and social relations, but also with feelings of guilt and illegitimacy. Conditions of importance for outdoor office work to happen and function well were found in the physical environment, where proximity to urban greenspaces stood out as important, but also in the sociocultural and organizational domains. Of crucial importance was managers’ attitudes, as well as the overall organizational culture on this idea of bringing office work outdoors. To conclude, if working life is to benefit from outdoor office work, leaders, urban planners and policymakers need to collaborate and show the way out.

## Introduction

In this age of urbanization, many people spend the vast majority of their time indoors, separated from the elements of nature, not the least at work. Why is that? Spending time outdoors and nature contact holds a great potential for various positive effects, both as prevention and treatment, upon well-being and health (Kuo, 2015; Frumkin et al., 2017). Historically, humans have spent most of their daytime outdoors, up to the dawn of industrialization, and the impact of constant indoor stay, in stressful work environments, is still not fully understood. Furthermore, contact with nature can contribute to improved executive functioning (Kaplan, 1995; Stenfors et al., 2019), recovery from stress (Ulrich, 1984), and boost in affective well-being (McMahan and Estes, 2015), with overall implications for cognitive functions and learning at large (Lisberg Jensen, 2009; Kuo et al., 2019). All these mentioned aspects are of relevance for tackling challenges in the working life of today, with its demands on social skills, problem-solving and creativity, possibly counteracting the negative effects of cognitively demanding tasks, and digital connectedness (Kompier, 2006).

Urbanization, resource exploitation, and lifestyle changes impact the possibilities for human contact with nature (Hartig et al., 2014). Remedies are suggested to bring more nature into the city or bring people out into nature (Turner et al., 2004). This study takes its point of departure in the potential of “everyday nature” (Kaplan et al., 1998), focusing on changes in everyday life in the context of work, possibly increasing (office-) workers’ contact with the urban outdoors, including the natural elements therein. The physical environment is a vital aspect often overlooked (Pratt, 2020) when work life is scrutinized for improvements.

The boundaries of physical spaces for work are also changing today and have become increasingly flexible (for some groups) (Allvin et al., 2013; Kossek and Lautsch, 2018), whereby work is being conducted at multiple locations outside the office, such as at home, in cafés, trains, and buses (Dale and Burell, 2008, p. 3). More and more office workplaces are designed as activity-based flex-offices, where employees do not have fixed workstations but share the office space with their colleagues (Bodin Danielsson, 2020). In such workplaces, employees are expected to switch between workspaces designed for particular activities, such as collaboration or concentration (Haapakangas et al., 2018). Aspects of these trends are far from unproblematic and especially lack of access to supportive facilities that enables personal control over unwanted stimuli appears critical (Bodin Danielsson and Theorell, 2019). It is not probable that tomorrow’s workplaces will look like yesterdays. As a matter of fact, the whole idea of traditional office buildings has been questioned with regards to the knowledge-intense working life of today (Duffy, 2008).

Despite increasing flexibility and boundarylessness, the threshold between indoors and outdoors seems to be high when it comes to office work. The office has a number of different functions at the social, functional, symbolic, and physical level (Söderberg, 2003), serving vital needs of people and their organizations by connecting colleagues, sheltering from wind and rain, enabling ergonomic seating/work positions, or offering desk-space and other necessary equipment. At the same time, an overreliance on indoor spaces is likely to be unsupportive of a knowledge-intense working life, where a growing number of people have non-routine, low-regulated works (Allvin et al., 2013). Many white-collar workers spend their workdays seated in office- or meeting room chairs, indoors, while a more flexible organization of the day may diminish sedentary behavior (Li et al., 2017).

Productivity is more intimately intertwined with our individual and intrapersonal capacity and ability to function, than ever. Ellström (2001) among others discusses the importance of a pendulum between different types of work-life learning and that opportunities for reflection and questioning of routines need to be incorporated into everyday work. Creativity is a wanted characteristic of employees, with creative output being something organizations crave. Does the modern office environment promote creativity? The lack of individual spaces and too much sound and movement distractions have been pointed out as a factor influencing creativity negatively (Stokols et al., 2002). On the other hand, the interactive social qualities of open plan offices can have a positive effect (Hoff and Öberg, 2015). A fruitful coupling is suggested between such insights about the prerequisites for creativity in work life with the evidence we have today on how natural surroundings can affect our activity, creativity, executive functions, social interactions, and health. Kuo (2015) describes how a “myriad of studies” have linked contact with nature to human physical and psychological health outcomes, including decreased prevalence of cancer, cardiovascular disease, and depression, through multiple pathways, such as natural sights, natural sounds, and negative air ions; physiological and psychological states, such as relaxation, awe, vitality, and attention restoration.

In addition, the outdoors, with regards to nature exposure, is interesting from a learning perspective (Lisberg Jensen, 2009; Sandell and Öhman, 2010; Mannion et al., 2013; Kuo et al., 2019). Kuo et al. (2019) list a set of direct effects of nature on the “learner” with nature rejuvenating attention, increasing physical activity, relieving stress, boosting self-discipline, and increasing motivation. In addition, they point out a set of moderating factors associated with the context based on evidence for vegetated settings contributing to a calm associated with overall feelings of safety and increasing the likelihood for fostering warm and cooperative relations.

The potential benefits of spending time outdoors and having more contact with natural elements in daily work life are of great relevance for meeting the challenges of today’s knowledge work. Incorporating outdoor office work practices may thus potentially contribute to a more sustainable working life. Attempts to test this have for example been done in academia, where walking seminars improved both perceived seminar quality and sense of well-being among participants (Bälter et al., 2018). However, studies are still largely lacking which investigate outdoor office work regarding possible practices in daily working life, what it may contribute to and its preconditions. Thus, the present study aims to fill this gap.

The overarching aim was to explore possible ways of working in the urban outdoors, as well as its potential benefits and challenges, to understand how such practices can contribute to a more sustainable and innovative working life. The more specific purposes were to: (A) identify and evaluate different forms of office work possible to bring outdoors – into the urban nearby surroundings; (B) understand how outdoor office work is experienced, and finally C) identify different types of contextual conditions of relevance for how outdoor office work is implemented and experienced.

## Materials and Methods

### Study Design: An Interactive Research Approach

The data collection took place within the frame of a two-year interactive research project, conducted in collaboration with a municipality in southern Sweden (September 2017–August 2019), and mainly funded by the European Social Fund. Interactive research is characterized by its collaborative approach in creating a change in practice, combined with a critical stance and development of theory (Svensson et al., 2015; Ellström et al., 2020). Mutual learning processes, across organizational borders, disciplines, and fields of practices are in focus (Johannisson et al., 2008; Ellström et al., 2020) to support the participants, as well as the researchers, in critically examining their own understanding (Svensson et al., 2015). Interactive research is part of the macro design rather than any specific research method or technique (Ellström et al., 2020). At the core is, “a two-way flow of problems and knowledge,” where not only the common, but also different interests of the participants and researchers are acknowledged. These activity systems can be seen as collective learning cycles producing common conceptualizations of the change process, through a joint analysis, aiming at going “beneath the surface” (Svensson et al., 2015, p. 353). Interactive research can be described as an approach within the action research family and, according to Eikeland (2007), the very core of action research is about radical self-reflection grounded in one’s own lived experience.

The project involved participants from the municipality making the actual changes in their work practice. The project organization contained a steering committee of directors; a steering group with managers from different departments; a project team of employees representing each group of participants, one project manager from the municipality and one from the university (the main author of this article). There was also a scientific board with six researchers from different universities and fields of expertise (the co-authors). In addition, there was the overarching steering from the European Social Fund.

**FIGURE 1:**
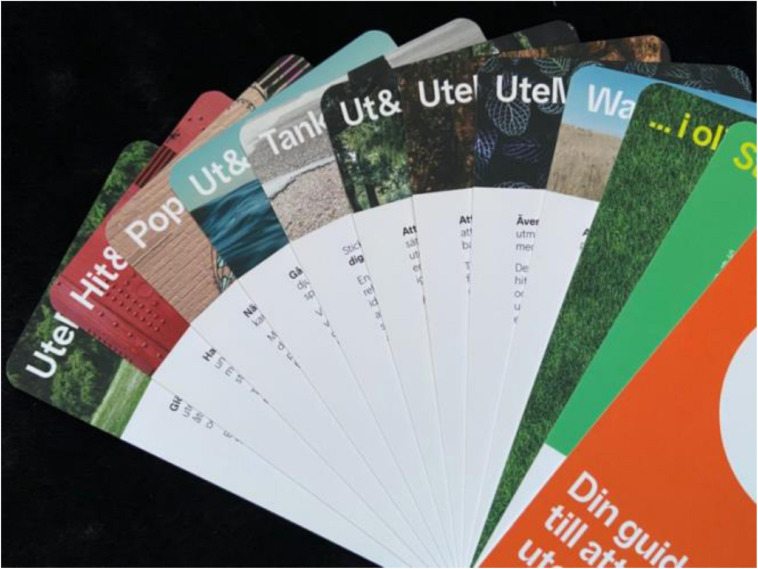
The pocket-booklet describing different forms of outdoor office work launched at the end of the interactive research project.

During the process numerous events were arranged in order to communicate and disseminate the knowledge from the project. During the final phase, a pocket-booklet (see Figure 1) summarizing the learnings from the project was produced and a municipality-wide week held with presentations, happenings, and a final conference with an exhibition at the City hall.

### Participants

The data collected, upon which this article is based, derive from participants who took part in the collaborative learning process, constituting the heart of the interactive research project (to be presented in further detail below). The participants were white-collar workers from five different departments within the municipality: The City planning office (*N* = 8), the Streets and parks department (*N* = 13), the HR-department (*N* = 9), the Cultural department (*N* = 16), and the Environmental department (*N* = 12). The participants were recruited on a voluntary basis and were initially reached through various information channels, such as presentations at department meetings, information at the organizational intranet, as well as many meetings with employees and managers spreading the word in their workplace.

From the start of the process 58 participants were enrolled including 14 men and 44 women from 27 to 65 years old. Apart from one participant, who explicitly wanted to be excused, another five fell out due to changes in their work situations (such as leaving their positions), while the others took part in the whole process. The professions represented included planning architects, administrators, HR-consultants, building inspectors, project managers, city planners, librarians, development coordinators, financial assistants, landscape architects, and communications officers. Experiences of a variety of workspaces were represented with participants having an individual office (14), a shared workspace for 2–3 persons (21), and a shared workspace for 4–15 persons (23).

### The Collaborative Learning Process and Qualitative Data Collection

**FIGURE 2:**
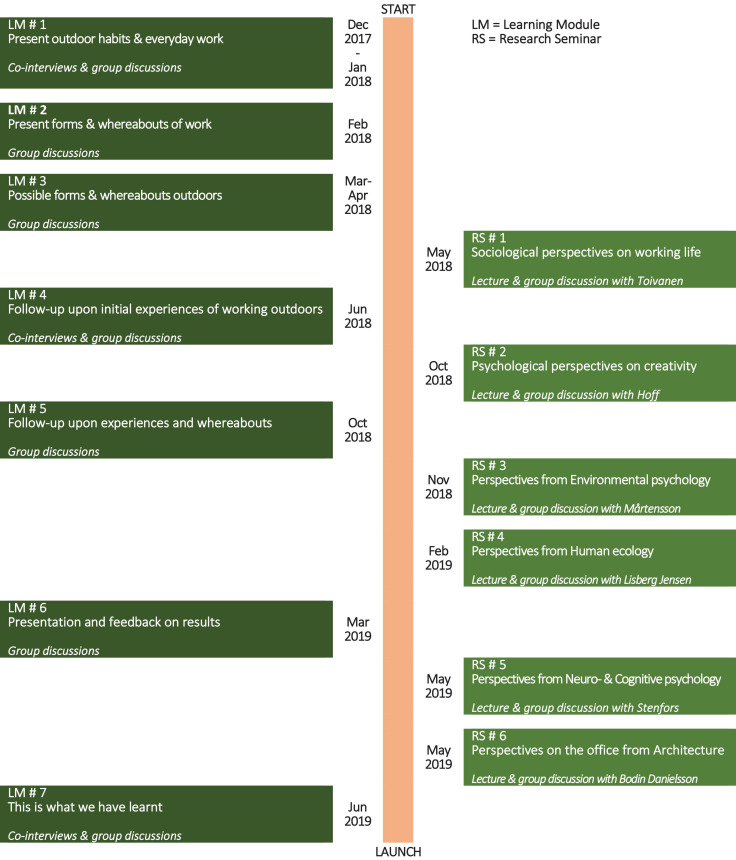
An overview of the learning process and the data generated.

The learning process was organized around seven so-called “learning modules” and six “research seminars.” *The learning modules* were arranged over half-days, with the First author of this article meeting up with groups of participants; usually five different groups with 6–14 persons in each. Most learning modules were repeated a number of times with each group of participants including numerous data collection heats in order to ensure everyone’s participation. In total 45 sessions encompassing qualitative data collection, took place. Each session entailed a check-in where everyone present commented their state of mind at the start, information-sharing about the project as a whole, followed by the processing of some specific questions and themes in reflective group discussions, alternated by various individual reflections and ideation-techniques and a final, individual check-out. In addition, “co-interviews” were conducted in the first and fourth module. This meant that the participants interviewed each other in pairs or threes, on the basis of a few preset, open questions. These, in total 51, co-interviews, as well as a total of 38 group discussions, were all recorded and transcribed. The first learning modules focused on the current ways and spaces of work. After that the participants’ self-managed, active experimentation and “testing” of bringing work outdoors took place during a 15-month period. The subsequent learning modules (from number four) were to follow up these experiences focusing on how working outdoors made them feel, what they needed and what kind of places they used and preferred. *The research seminars* were made up by an introductory lecture, where the researchers involved (the co-authors) presented their research perspectives. The seminar continued with a group discussion, preceded by a “walk-and-talk” in smaller groups, about what had been learnt and could be taken into account for the process of exploring outdoor office work onward. See Figure 2, for an overview of the formalized learning process and their themes, as well as the data generated.

**FIGURE 3:**
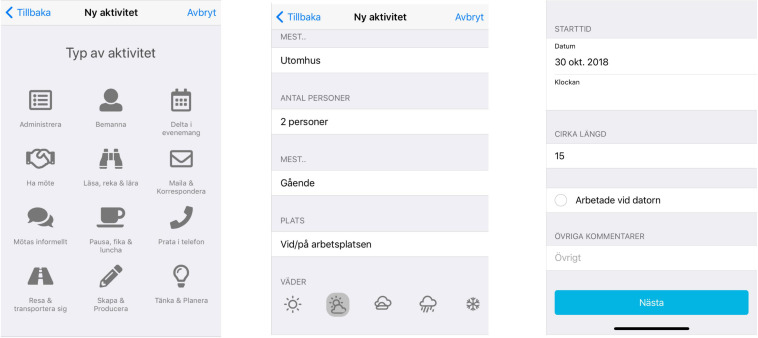
The interface of the mobile app.

*The main data*-set contains material from co-interviews and group discussions during the different learning modules. There was also other material collected during the process which was helpful to further our understanding: A *mapping* procedure was to create an awareness among the participants about their work situation, to unbundle their work activities to see which possible parts could be tested/taken outdoors. *A mobile app* developed in dialogue with the participants enabled them to log their outdoor work endeavors and experiences. The app included a number of descriptive elements of the work situation outdoors including what kind of work activity, but also how many people were involved; whether it was mainly sitting down, standing, walking, or other; what the weather was like; what kind of outdoor setting (outside at the workplace, a park, a square, streets, etcetera); approximate time interval; in addition, there was an open space free for comments (see Figure 3).

In total over 700 individual outdoor work activities were logged, unevenly distributed among the participants. Many participants had a hard time keeping up the logging, as the outdoor activities gradually became more and more habitual.

### The Process of Analysis

The learning cycles of interactive research entail continuous joint processes of analyzing (Ellström et al., 2020). The procedure with the learning modules and research seminars, and the participant’s own, active experimentation in between, was set up and conducted in order to facilitate experiential learning (Kolb, 1984). Thus, the “real-life” experiences in the participants’ everyday work setting were central, but so were the common reflections, discussions and conceptualizations, which sought to catch, analyze and draw conclusions from the same, by making the implicit, explicit. The process of analyzing aimed at making everyone more aware of the underlying values, which govern actions (Argyris, 1976). Eikeland (2006b) points to the need to provide space for learning, and to facilitate “learning-to-learn”. He highlights the importance of an alternation between activities related to “on-stage performance”, where tasks and roles are performed in the work organization, on the one hand, and “back-stage reflections”, where experiences can be critically analyzed and discussed, on the other. There needs to be an arena for encounters and dialogue between the researchers and participants, in order to organize the processes of collaborative inquiry essential to interactive research (Ellström et al., 2020). In the present project the learning modules filled this function. From a research point of view, the repetition of the learning modules gave an opportunity of collaboratively analyzing, synthesizing, questioning, altering, and validating the preliminary findings, along the way. That is, during the process, the experiences of conducting work outdoors documented in co-interviews and group discussions, were also analyzed in a step wise procedure of qualitative analysis (Holsti, 1969). This focused on identifying statements referring to more positively and negatively valenced experiences, emotions, and feelings. Statements could occur in a variety of forms, such as a word, phrase, sentence, or paragraph. Categories derived from the empirical material have been further conceptualized after the end of the interactive phase of the research study.

The log kept in the mobile app have mainly served as a complement to the aspects brought up in the group conversations and co-interviews of the learning modules and was of value, especially for broadening the picture when it came to the identification of different forms of outdoor work.

### Ethics Statement

The project was conducted in accordance with Swedish national legislation. All data collected has been anonymized when presented and the data in the mobile app was untraceable to any certain individual. Participation in the project was voluntary and participants gave their informed consent. In the context and practice of action research, there are multiple ways of understanding and enacting ethics and while a further elaboration upon these falls outside of the scope of this text, some important elements and implications are discussed by Brydon-Miller (2012) as well as by Eikeland (2006a).

## Results

The results will be presented in accordance with the three main questions guiding the interactive research project, about different forms of bringing office work outdoors, how it is being experienced, as well as what conditions it requires that are of relevance for outdoor office work to happen and function well. A tree may be envisioned as a tentative metaphor for the various results: The main branches are then different forms of doing office work outdoors; the fruit illustrating what experiences outdoor office work may bring about; the soil representing the sociocultural dimensions, within which the structural organizational conditions – the trunk, that is – need to be firmly supported through its roots; and finally, there is the surroundings, illustrating the physical environment as a context for outdoor office work.

### Unbundling Opportunities for Action

At the outset of the learning process, doubts were expressed by participants as to whether it would be doable to bring any substantial amount of work activities outdoors. The hesitation was mainly attributed to the fact that they needed various things at their workplaces and most of all they thought about the dependency upon their personal computers and access to ICT-systems for nearly everything – and that it probably would not work well to bring it/their laptops with them, outdoors.

Against this background, the mapping of participants’ activities during an ordinary work week served an important purpose in unbundling the work and opening up for finding opportunities of bringing some of it outdoors. More concretely, mapping resulted in the following twelve typical work activities being identified:

• Thinking and planning (solving problems, getting ideas, analyzing, structuring thoughts, etc.)
• Creating and producing (texts, drawings, presentations, other material, and artifacts)
• Talking on the phone
• Participating in events (workshops, conferences, courses, etc.)
• E-mailing and corresponding
• Administrating (reporting, ordering, etc.)
• Reading, scouting, and learning (activities that were about “taking things in”)
• Holding/attending meetings (planned, formal ones)
• Meeting informally (unplanned, smaller ones, spontaneous chats included)
• To man/crew (such as the helpdesk at the library)
• Traveling and transporting oneself (back and forth to different meetings and events)
• Taking a break, lunch break or “fika” (Swedish word for tea or a coffee break)

These categories were, and are not, to be regarded as stand-alone concepts, but rather as a working material facilitating the planning and communication of the outdoor work activities, also making up a framework for the experience registering in the mobile app. Thus, the main point was for the concepts to be clear and agreed upon among participants and researchers. Not far into the process, reflections about the current situation, the mapping and the planning that took place during the first four months of the process (learning module 1, 2, and 3), led up to a sense of heightened awareness among the participants, regarding what they did, what they needed, appreciated – and not – in their habitual whereabouts of work.

### Forms of Outdoor Office Work Identified

Based upon the participants’ active experimentation of bringing parts of their typical work activities outdoors, logging in the mobile app, as well as a continuous follow-up along the process, nine forms of doing work outdoors were identified and categorized into four main kinds: collaborative work activities, individual work activities, outward-facing work activities, and in-between work activities.

#### Collaborative Work Activities

##### Walk and talk

Taking a walk in order to talk was one of the most popular work activities to do outdoors. This most commonly used outdoor form of work is – more or less – well known and spread before and outside of this exploration. It was perceived as easy to make happen, as it did not require any special plans or arrangements. All that was needed was a nice, preferably green and calm enough path for walking, in proximity to the workplace and some comfortable shoes and clothes suited for the weather. These were appreciated for breaking up an otherwise sedentary workday, and for contributing to feelings of enhanced openness and ease in the conversations, as this participant expressed it:

*“I feel that the conversations become really good, especially when one walks and moves about (….) It is as if something extra happens*….”

The “Walk and talk” was usually taken in pairs, or maybe in threes, but could also involve more participants when constituting parts of bigger meetings and events, whether held indoors or outdoors, as in the next form for outdoor office work.

##### Outdoor meeting

Not only meetings in motion were performed outside, but also regular “sitting meetings,” as well as more formal and extensive ones. “Outdoor meetings” could be organized much like indoor meetings but were conducted under bare sky (or under a pergola). It could be a couple, or a group of colleagues, locating their meeting to the inner courtyard, or just in front of, their workplace, or elsewhere in the surroundings, as in a park. Clearly, the smaller meetings were closer at hand, as this participant comments:

*“Often, meeting two by two has been the easiest, where you do not need so many aids. If one is to go things through, demanding that one has access to a lot of files and such, then it has been easier to do it inside. But sometimes you mostly need to sit down and talk something though, or plan something and then it has worked well to take it outdoors.*”

Outdoor meetings could however also be various forms of department- and unit meetings, project meetings and workshops, as well as meetings with external collaborators and guests. Sometimes these meetings were organized very much in the same way as indoor meetings with people mainly sitting down. At other times they also contained walk and talks, to and in a nearby park, as well as standing and sitting gatherings. In one case, it was all prepared with an overall program, a map and particular questions for the various sessions. When it came to these bigger and more formal outdoor meetings it was clear that they demanded more preparation and planning than the ordinary (indoor) ones. However, even so, they showed to hold a potential, as commented upon by one participant:

“We have had one (…) but, I actually think that we can have more departmental meetings outdoors. Because everyone was really gratified.”

#### Individual Work Activities

##### Outdoor office

One form of bringing work outdoors was that of participants simply going outside by themselves, sitting down, doing things basically in the same way as inside of the office places, with or without their laptops.

Participants’ views upon using the laptops while practicing “Outdoor office” were two-tiered. One, meaning that laptops should be left inside, either because the quality of the visuals/the screens were not sufficient, or because they stated that detaching themselves from the computer and constant connectedness was the whole point of going outdoors. The other, finding it quite alright to work with the laptop outdoors, as long as the screen was good enough and as long as it was possible to find a place to sit down, with sufficient shade under a parasol, awning, or the like. A few stated that they were so dependent upon their computers and ICT-systems for everything that they would have a hard time to find ways of working outdoors at all, if they could not make it work with their laptops, which was the case for this participant:

“I started by sitting with sun-glasses and the computer, but realized that it did not work, so I folded up the parasol and then I could take my sun-glasses off. Just enhanced the light on the computer and then it went perfectly well. I think that I sat there for three, four hours.”

Some form of roof protecting from sun and rain, as well as a place comfortable enough to sit down and, when bringing it – a table for the laptop, were some of the obvious needs brought up. One also frequently mentioned aspect was that of wanting something behind one’s back when working to avoid the unwished-for feeling of being watched from behind.

##### Outdoor reading

Bringing something to read, or go through, outdoors was perceived and described as an especially simple and intuitive way of breaking a piece of work out and thereby making outdoor work happen. Therefore, it was found to deserve a form of its own, even if it could be seen as a variant of doing “Outdoor office.”

##### Think walk

Having a walk, in order to think, was another way of conducting work outdoors. The purposes were many and varied among participants. They took a “Think walk” in order to structure their thoughts, to plan, to analyze, to reflect, and solve a particular problem or to just let their mind wander, get inspired and develop their ideas. Just like with “Walk and talk” the main needs in connection to this form of outdoor work was access to, and knowledge about, good walking paths and routes, away from traffic and other noisy and potentially hazardous elements.

#### Outward-Facing Work Activities

##### Outdoor Learning

This form for outdoor work has a well-known base and sibling in outdoor pedagogy, in educational settings. In this context – of work, it was mainly seen as a way of experiencing things and places in the city, at first hand – and was used both individually and in groups. “Outdoor learning” became somewhat of a reminder that there may be good reasons to question the increasing habit of (always) turning to information, pictures and maps on the internet, instead of leaving the office to get a talk with people and to see and experience different sites and artifacts for oneself, when possible. This form of outdoor work included various types of field trips and study visits and may contribute not only to learning, but also to the development of relations, as this participant described:

“Yesterday, we had a unit-trip (…) where we walked and saw different things (.) and were outdoors. And it was so much energy that it gave and joyfulness and feeling of community. So, I believe in outdoor activities, where one experiences the same things and not just sit inside four walls.”

##### Pop out service

The typical work activity “to man/crew” – that is, to be available at a certain place and responsible for some service to the public, may be seen as impossible to bring outdoors. However, some participants actually took their operation outside of the building, or on a tour. The examples were not many, but still a potentially important way of moving work outdoors. One of the workplaces was a library situated in an area where shootings were a recurring hazard. There, they brought their helpdesk – i.e., themselves, on a blanket – out, in the middle of the square right outside the premises of the library. By doing so, they found that people who did not normally come inside to the library, met up and joined their activities. Thus, lowering the threshold to interact with the organization and contributing to a sense of community and safety.

It may be added that the outward-facing dimension showed to be present in other forms of outdoor work as well, as spending time and moving about outdoors led up to unexpected and unplanned for meetings. One of the participants, who had access to one of the most favorable outdoor areas right outside the workplace regarded this as a positive aspect of it and described how individual outdoor work activities or meetings can become a form of Pop out service:

“Due to the fact that we have begun to spend more time outdoors, it has led to more informal meetings. So, we have actually become a kind of, not attacked I should say, but actors move about in the area and then they have seen us and thereby started conversations, also which have become new meetings, in a positive way. Whom, if we would have been sitting indoors, we never would have encountered. So, in some way it makes us visible in the neighborhood where we operate.”

#### In-Between Work Activities

##### Outdoor transportation

Transporting oneself – going from here to there, was an integrated part of many of the participants’ work week, which almost *per se* meant some contact with the outdoors. Still, the project participation led to the logic of getting to places in the fastest possible way (by car or bus) being questioned. The possibility and point of instead using some kind of “Outdoor transportation,” such as going by bike or walking was raised. This change of mode in some cases turned the transportation into “Walk and talks,” and even “biking meetings,” in a couple of cases.

##### Outdoor break

Going outdoors when having a break was the most common way of getting outside of the office building during the workday, both before and at the end of the project. However, the practice of taking an “Outdoor break” became accentuated and lifted as more important than before.

To sum up, the described forms of working outdoors were embraced and used to varying extent among participants, possibly due to a diversity of a number of factors, such as personal preferences, workplaces, outdoor environments, work roles, and professions. Some activities were closer at hand and occurred more frequently, such as “Walk and talk” and “Outdoor reading,” while others were more dependent on weather and other attributes of the physical environment, especially those performed sitting down for a substantial amount of time. However, every one of the typical work activities identified to be possible to do outdoors were practiced by some participants, though more or less frequently. A common reflection among participants was that the process made them conscious about their room for maneuver. Consequently, despite the initially anticipated limitations, after the 18-month learning process of reflection, active experimentation and follow-up discussions, it was evident that a wide range of work activities could be brought outdoors and serve several purposes, both individually and together with others.

### Experiencing Outdoor Office Work

How did participants experience working outdoors? The qualitative thematic analysis of the group discussions and co-interviews provided a more detailed understanding of how participants experienced their outdoor work as rewarding and/or problematic. The analysis identified 12 subthemes that were organized into five main themes. The participants experienced wellbeing and recovery, autonomy and distance, enhanced cognition, improved communication and relations and finally also some inner resistance and illegitimacy.

#### Well-Being and Recovery

##### Feeling good

When asked to recollect how spending time outdoors in general made them feel (during leisure-time, vacations or in everyday life) participants frequently expressed things like “it feels good” or “great” and the same adjectives continued to come up frequently when asked about how they experienced working outdoors. Apart from terms such as “good” and “great,” words like “lascivious,” “happiness,” “lively,” “lovely,” “enjoyable,” “very, very nice” were prevalently used.

##### In contact with nature

Another very prevalent theme was a feeling of noticing and being in connection with the shifting outdoor environment, especially with nature phenomena. Participants expressed that it was nice to see daylight, feel the grass, the scents, the sun, the heat, the sounds, that they felt like one with nature (and even the seagulls!).

##### Energized

Feelings of alertness were also frequently mentioned. The participants stated that they felt more alert, got more energy, better endurance, focus, and concentration – also spilling over onto when going back indoors. Some participants also mentioned feeling more productive, when working outdoors.

##### Calm

This theme comprises many expressions for a feeling of becoming calmer, that it was perceived as de-stressing, but also much about the need to let the many, constant impressions out, rinsing ones’ head, get rid of bad energy, as well as comments about better sleep after spending more time outdoors.

#### Autonomy and Distance

##### Free and empowered

Many were those mentioning feelings of freedom and autonomy, in terms of “when outdoors I totally decide for myself” and also comments about that it was a good feeling to get/feel trusted by the managers, as some of the participants did, while others did not. One of the latter expressed that she more and more felt like “an animal in a cage,” when sensing that it was not accepted to leave the office for an outdoor work activity.

##### Given a chance of getting away

This is about the chance of getting away, getting some distance and sometimes being on one’s own, not being disturbed, disrupted, and getting a change of environment.

##### Able to breathe

Working outdoors was mentioned as a way to get oxygen and being able to breathe, both in a literal and more symbolic sense. There were also examples of participants taking a deep breath, when trying to recapture what the outdoors made them feel.

#### Enhanced Cognition

##### Able to think

This theme was made up by many comments about the outdoor work activities giving concentration and better focus, especially the “think walks” gave space for thought, such as mental preparation, reflection, for thinking things through, solving problems, structuring, and batching work activities and assignments.

##### Inspired and creative

The feeling of being inspired and more creative was often expressed in those very words, but also as seeing other things, new perspectives, new ideas and generally opening the mind up.

#### Improved Communication and Relations

##### More open and equal conversations

Descriptions of more relaxed situations, more laidback, more open and deeper conversations and a different, better dialogue were reoccurring. And also comments about other relations and new constellations, a sense of community by experiencing common things, as well as perceptions of more equal relations. These dimensions were often brought up in relation to experiences of walking (beside one another) and talking, both when it comes to the openness of the conversations and to a lessened presence of hierarchies. One participant felt that she had a hard time finding a good way of describing it, but put it like this:

“When you walk together you have a different type of dialogue than when you sit opposite from one another and talk (….) I think that it becomes a kind of, I don’t know, equal, or that you – I, at least – feel that one gets a deeper dialogue.”

#### Inner Resistance and Illegitimacy

##### Difficulties in changing habits

The participants experience some difficulties and more or less practical hindrances for bringing work outdoors, but most of all they mention the difficulty in changing habits.

##### Guilt and expectations from others

Spending time outdoors while working evoked feelings of guilt and many were the comments on the illegitimacy of “sitting outdoors – enjoying oneself.” They also pondered about whether it was themselves mainly, or others (managers and maybe colleagues) who upheld these norms. This is how one participant expressed the emotion:

“I do the very same work-task as I would have indoors. But, I feel a bit guilty. As if it is a bit mischievous to go outside. And I don’t know why. Is it me thinking like that?”

Apart from this explicit reflection about where norms reside, there were many comments about their existence, for example expressed in terms of: “I feel a bit guilty,” “frivolous,” “as if one escapes,” “I feel resistance from my inner norms,” “there is an unwritten rule that you do not work outdoors.”

### Conditions for Outdoor Office Work

One of the most prominent possible obstacles to working outdoors, brought up by the participants at the beginning of the learning process, was that of the Swedish weather – that it would be too cold, rainy or too windy most of the time, for outdoor office work to be attractive and well-functioning. As mentioned previously, participants also anticipated that it would be difficult to be away from the office, not having direct access to various work-material and technology – especially their own computers and ICT-systems. However, even if these were factors of importance for what, when, how often and with what ease work activities could be pursued outdoors, there were other factors emerging as even more cardinal; both when it comes to physical, as well as sociocultural and structural organizational conditions.

#### Physical Conditions

##### Proximity to a pleasant outdoor environment

For outdoor work to happen at all, access to an attractive and well-functioning outdoor environment was of utmost importance. It basically needed to be right outside the workplace, if it was not to be experienced as inconvenient and/or too time consuming to alter the normal indoors with outdoors. Depending on work activity, different types of settings were needed and appreciated, however with some familiar characteristics of being green, lush, and tranquil enough. Secluded places were wanted for sitting down by oneself, or when gathering for an outdoor meeting, while a “think walk” or “walk and talk” required nice and easily accessible walking paths, on safe distance from traffic and noise.

##### Infrastructure fit for the purpose

Outdoor work encompassing sitting down, such as “outdoor office,” “outdoor reading,” and “outdoor meeting,” required some infrastructure and artifacts designated to support the activities they had planned for. These included both physical, technical and/or symbolic ones, such as shields for rain-, wind-, and sun, meeting arrangements, comfortable seating and appropriate tables, some kind of screen/board for sharing visualizations, and also access to Wi-Fi, power outlets and, also things like blankets and coffee. Outdoor office arrangements were also asked for to legitimize outdoor work activities, just like “walk and talk-maps” were wanted both for practical/informative and more symbolic reasons.

#### Sociocultural and Structural Organizational Conditions

##### Apt and clear policies, rules, and regulations

There are rules and regulations to consider related to outdoor work, such as work safety, insurances and health, as well as HR-policies, management- and reporting-systems. Drawing upon the question-marks brought up by participants, now and then, throughout the learning process, there was an ambiguity surrounding these formal issues as to whether they, in their existing forms, actually admitted this kind of flexibility becoming institutionalized, or not (after and outside of the interactive research project, that is).

##### Leadership based upon trust

Furthermore, the perceived attitude of managers toward flexible working conditions in general and the idea of bringing work outdoors specifically, were found to be of great importance for the participants as a barrier or enabler. The value of managers themselves suggesting, for example, a “walk and talk,” could not be overstated, from a policy perspective. Another most important dimension according to the experiences of participants was that of leadership style; whether it was predominantly based upon a need of (direct) control, or trust. Leadership built upon trust, admitting room for action and autonomy in the everyday work situation, was seen as absolutely essential.

##### Open-minded and supportive culture

Culture was a topic frequently revisited, more and more often, as the learning process proceeded. The perceived attitude and culture in the closest work group was of importance for participants, whether they felt that it was accepted or not to leave the office for a while. One participant expressed it like this:

*“I reflected a bit upon what it is. Why your colleagues a kind of look twice when you are on your way out. Is it that they think that one is more ineffective when going outdoors. What is one afraid of? That you will shirk work*… *that you will not be as effective?”*

Also, the overall organizational culture was discussed and brought up as an important factor in this context. Values and norms about work emerged as one of the – if not the one – most important aspect hindering outdoor work activities to happen and/or be incorporated into everyday working life. With reference to the feeling of guilt, witnessed and shared by many, participants asked themselves and one another: How pleasant is work allowed to be?

As shown above, there are both elements in the physical setting as well as sociocultural and structural dimensions of importance to understand and develop, when integrating urban outdoor spaces into everyday working life. Thus, there are barriers and challenges of different kinds to overcome. Basically, all of the mentioned facilitating factors and needs can be regarded as crucial in order for outdoor office work to take place, be attractive, well-functioning and beneficial, while the lack of them, or their opposites were brought up as factors standing in the way.

## Discussion

The general results of the presented project show that there are pleasant experiences from spending time outdoors and having contact with nature also in the context of office work. Benefits from being outdoors have been found in many other studies in other contexts (Mangone et al., 2017; Twohig-Bennett and Jones, 2018).

The findings of the first research question about *identifying work forms*, was that nine different work activities may be brought outdoors for various purposes, both individually and together with others, for activities facing outwards (as when providing the service outdoors) and for transporting oneself or having a break.

The second research question aimed at understanding *how outdoor office work is experienced*. The findings show that when working outdoors participants experienced predominantly positive feelings of wellbeing and recovery, autonomy and distance, enhanced cognition, improved communication and relations, but also feelings of guilt and illegitimacy.

The findings of the third research question about *identifying contextual conditions* of relevance for outdoor office work clarified that proximity to attractive outdoor greenspaces and walkable surroundings are important for outdoor work to happen and moreover some physical facilities may be needed for it to function well, such as furniture, wifi and sun-/rain-/windshields. In addition, sociocultural and structural organizational conditions related to policies as well as norms about where work should take place were central. An organizational culture and a leadership that facilitated outdoor work was decisive for the ability of developing such work habits.

### Well-Being and Functioning in a Knowledge Intense Working Life

The knowledge intense and boundaryless character of today’s office work (Kompier, 2006; Allvin et al., 2013) entailing high cognitive demands, means that cognitive capacities are vital in order to manage work. At the same time, a high load of intellectual work demands can increase the risk of cognitive stress and stress related mental health conditions, commonly characterized by cognitive dysfunction in the domains of executive functions and memory which are important not least in order to manage and perform knowledge work (Stenfors et al., 2013a, b). It is as such imperative to develop and enable the utilization of workspaces and work practices which support and replenish these cognitive capacities and support mental health and wellbeing. In the present project, results show that enabling and incorporating outdoor work among office workers/employees can serve such purposes (i.e., support different aspects of cognitive and mental health and functioning) and thus support a more sustainable work life, provided that qualitatively suitable outdoor spaces were available in close proximity to the workplaces. Some of the key qualities sought for in an outdoor workspace were greenspace, tranquility, and walkability. More specifically, outdoor office work was by the participants experienced as beneficial from the aspects of wellbeing, for restoration of cognitive capacities, and for enabling distance to cognitively and emotionally taxing contexts. Outdoor work opportunities thus appeared to play a role in freeing up cognitive resources required for more demanding problem solving, planning, and processing, as well as allowing an expansion of the mind in terms of reflective thinking.

The experienced benefits of working outdoors expressed by the participants corroborate the systematic findings of previous controlled studies on how especially outdoor nature contact can facilitate cognitive restoration and better executive cognitive performance (Stenfors et al., 2019), in line with Attention restoration theory (Kaplan, 1995), which also support reflective and creative thinking (Williams et al., 2018).

However, it is worth noting that optimal environments with regard to performance on standardized tasks may differ between demanding executive cognitive tasks – which are more sensitive to stress and cognitive overload via e.g., distractors/stressors in the environment (e.g., Shields et al., 2016) – versus monotonous tasks with low executive load. For example, Jiang et al. (2021) found that performance on a simulated driving task during a prolonged time (90 min) was optimal in a simulated freeway landscape with moderate levels of greenness and more complexity, rather than higher greenness and less complexity. That is, for such types of long-duration monotonous tasks, the environment should provide an adequate level of stimulation (that is, not being over-relaxing) to keep an adequate level of arousal, according to the authors, in order to perform the task optimally. Arousal *per se* was however not measured in the study.

It is furthermore worth noting that the optimal level and type of environmental stimulation when performing different tasks is individual and context dependent, where the sense of personal control is important (Shields et al., 2016; Tsai et al., 2019). Hence, the individual’s control over the physical and social working environment is vital, as discussed further below.

The boosts in wellbeing and feelings of connectedness with nature, which participants experienced while doing office work outdoors (feeling good, energized, calm etc.), also mirror the positive effects of outdoor nature contact found on different aspects of positive affective experiences in prior work (e.g., Kuo, 2015; McMahan and Estes, 2015). Furthermore, outdoor office work was reported to enhance the social climate and different types of communication (open and equal conversations) with a positive bearing on both the work itself, as well as social relationships – another well-known key factor to a health-supporting work situation (e.g., Stansfeld and Candy, 2006).

### Personal Control

The beneficial experiences of outdoor office work could be interpreted also from a job control perspective, as conducting work outdoors might give an increased sense of control. As the participants expressed under the themes “wellbeing and recovery,” and “autonomy and distance,” working away from unwanted taxing stimuli in the office gave opportunities for recovery and to be undisturbed. It is well-documented that the possibility to withdraw in order to concentrate or have a private meeting is an important aspect of job satisfaction (Bodin Danielsson and Bodin, 2009; Bodin Danielsson and Theorell, 2019). The general urge to gain some sense of control in the work situation is often related to the combat of different indoor stressors, but applicable also to the evaluation of the surroundings regarding its feasibility, accessibility, and pleasantness (Lindelöw et al., 2014). The major environmental stressors in open office environments are visual and sound disturbances (Sundstrom, 1986; Jahncke et al., 2011) and crowding (Langer and Sægert, 1975). They have negative impacts on various outcomes, e.g., employees’ health and well-being (Dean et al., 1975; Wallenius, 2004).

The project participants experienced outdoor work to facilitate “enhanced cognition” in several ways, which can be contrasted with the fact that indoor open plan offices have been shown to have negative influence on employees’ ability to concentrate and be productive (De Been and Beijer, 2014). Having a sense of control is in stress research considered to reduce stressful events (Lazarus, 1966). At work the individual’s control depends on various factors, for example on the ability to plan and execute work activities (Karasek and Theorell, 1990), but also on control over the physical work setting. Among office workers it has been found that the individual’s sense of control over physical aspects of the physical environment mediates the relationship between perceived distractions and perceived job performance (Lee and Brand, 2010), something which might be a reason for expanding possibilities for outdoor office work in the future.

### Creativity and the Need for Space and Inspiration

Creative performance appears also to benefit from outdoor work as expressed by the participants (“inspired and creative”). The theme “autonomy and distance,” which also relates to the need for control (and solitude) is another example of how outdoor office work can play a role for creativity. With many employees sitting in open plan offices, the lack of spaces for solitude, reflection and silence might hinder creative thinking (Stokols et al., 2002; Hoff and Öberg, 2015).

The participants’ experiences of working outdoors can in several other ways be related to what is described as conditions for creativity, which include both psychosocial and physical aspects. Organizational psychologists have stressed psychosocial climate aspects such as, freedom, openness and playfulness as some of the essential dimensions of a creative work climate (Isaksen et al., 1999). Similar conceptions were found in the participants’ descriptions of outdoor work, for example in the subthemes, “free and empowered,” “open and equal conversations,” and “inspired and creative.” A fourth dimension stressed by Isaksen et al. (1999) is idea time, and that could be connected to the “autonomy and distance” theme.

Just like the participants reported that outdoor work made them feel “inspired and creative,” research literature has demonstrated that physical outdoor aspects affect the creative process in several ways (Atchley et al., 2012; Oppezzo and Schwartz, 2014). Oppezzo and Schwartz (2014) found in an experimental study that participants who completed several types of creativity tasks outdoors outperformed those who did the tasks indoors.

The response theme “wellbeing and recovery” goes in line with another part of the creative process, a phase in which slowing down is essential. The creative process consists of several phases (Cropley and Cropley, 2010). After problem definition, a phase called incubation takes place, which means taking a break from active work and shifting to less focused cognition. During incubation inspiration is sought and unconscious processing takes place (Dijksterhuis and Meurs, 2006), something which helps the creative team before deciding which ideas to implement. Some part of the outdoor working time of the present participants might have functioned as incubation.

### The Landscapes of Outdoor Office Work

For outdoor office work to happen and function well there needs to be access to the particular “affordances” office-workers look for, the attributes which make it feasible to carry out their tasks and the characteristics of a landscape which make it worthwhile to move their work outdoors.

Several of the identified positive experiences of outdoor office work in the present study, which are important for well-being and functioning in a workplace context have also been highlighted from a design perspective. For example, Ulrich’s Supportive Design Theory (Ulrich, 1991, 1997) suggested primarily three health supporting design characteristics which serve to reduce stress and enhance recovery. These characteristics include those that facilitate and enable a sense of control, social support, and positive distraction (Ulrich, 1991, 1997). Jiang et al. (2019) further proposed to add the health supporting characteristic of facilitating and providing opportunities for physical activity. Relating to Supportive design theory, the current study findings suggest that the availability of attractive outdoor spaces (including greenspace), is an important factor for people to ‘get out’ during the workday and through this get more physical activity and stay less sedentary during the workday. Furthermore, well designed outdoor spaces around the workplace also serve to enhance the opportunities for a personal sense of control (i.e., providing more spaces to choose from), social relationships and support, as well as providing positive distraction (Ulrich, 1991, 1997; Jiang et al., 2019).

The overall challenge of planning and design would be how private, semi-private and public places, part of the cityscape in the surroundings of office buildings, can turn compatible with workers’ multidimensional aspirations to good workspace, including diverse practical requirements in line with their work tasks. Shelters of wind, sun and eyesight from behind, are some of the basic requirements and if the sites in addition to this are lush, green and tranquil they might be able to create an “outward pull” in the daily life of the organization, making the staff motivated to make some effort to move some of their work-tasks outdoors.

What we know from earlier research is that there are combinations of green and built elements which together form the urban sites commonly preferred and housing the restorative qualities (Kaplan et al., 1998; Tenngart Ivarsson and Hagerhall, 2008) which people tend to look out for. Such “comfortable design” emphasizes the easy access to an outdoor environment containing enclosures, conveying feelings of safety and with good possibility to visual orientation (Bengtsson and Grahn, 2014).

However, the participants also pointed out the role of more active interaction with the built and green cityscape when they mentioned pleasant nature scents and noisy seagulls, while having an “outdoor meeting” in the inner courtyard, or during active transport to destinations in other parts of town. This highlights the possibility of practicing a more place-responsive approach (Mannion et al., 2013) to outdoor office-work in the future, in which the surroundings are more actively used to stage an outdoor workday by drawing on the resources of people and places; for example, by arranging outdoor meetings, instead of or in combination with digital communication.

Participants’ feelings of vitality and being energized by outdoor office work may be associated with having contact with nature, but could also be due to being more active at large (Roe and Aspinall, 2010). The combination of physical outdoor agility and mental agility during outdoor stays might facilitate social, self, and emotional regulation processes in the transactions with place (Korpela et al., 2001; Mårtensson et al., 2009). A unique cityscape with its green, blue and built environment can contribute to attractive destinations and places in which people can stage meetings or find room for episodes of social or solitary work. This is well in line with the walkability literature discussing how urban temporal policies can be developed to get walks integrated into the everyday lives of urban dwellers by making the city, not only feasible to cross, but create routes that are safe, comfortable and even pleasurable (Lindelöw et al., 2014).

The compatibility of available outdoor spaces with the needs and current work task demands is a complex issue intertwined in a more general question of how to create livable urban environments. In accordance with the Urban Agenda Platform (2020) the planning for outdoor spaces needs to go hand in hand with the development of urban biodiversity and other ecosystem services (Gunnarsson et al., 2017; Hedblom et al., 2017).

### Limitations and Future Research

There are some limitations regarding the present research, such as the relatively limited number of participants involved in the testing of outdoor work possibilities. The participants came from several different departments, but they all worked in a municipality in southern Sweden. Future research needs to investigate whether the nine forms of outdoor work activities identified in this study need to be expanded in contexts with other cityscapes and weather conditions. Applying other approaches could give clues of the effects of outdoor office work. The experienced benefits of working outdoors should be corroborated systematically, to better understand their impact on the employees. Finally, research needs to further investigate the physical as well as sociocultural and structural organizational prerequisites of office work to better understand how outdoor work environment, leadership and culture may foster outdoor office work and overcome obstacles, such as indoor habits and norms.

## Conclusion

The present study contributes with new insights into how outdoor office work may be done—what works well versus less well; how employees experience outdoor work – benefits and hinders; and what conditions are necessary to fulfill in order to make it happen.

According to participants’ experiences, outdoor office work appears to support wellbeing, recovery, autonomy, enhanced cognition and communication which in their turn might play a role in work productivity. It seems to offer employees a strategy to increase personal control and give support to different aspects of cognitive and mental health, functioning, and creativity as well as improving social relations. Outdoor office work can thus contribute to and support a more sustainable and innovative working life, provided that qualitatively suitable outdoor spaces are available near the workplaces. Some of the qualities sought for in an outdoor workspace were green and lush places to get tranquility for sedentary work and walkability for work in motion. The conditions also included facilitating sociocultural norms and structures to encourage outdoor work.

The results of the study indicate how outdoor office work, in many different ways and dimensions, can contribute to a more sustainable working life. Furthermore, the barriers to a more productive and health promoting outdoor work life appear to be more related to sociocultural factors, than to practical issues. It is therefore imperative that leaders, urban planners and policymakers collaborate and show the way out.

## Data Availability Statement

The raw data supporting the conclusions of this article will be made available by the authors, without undue reservation.

## Ethics Statement

Ethical review and approval was not required for the study on human participants in accordance with the local legislation and institutional requirements. The patients/participants provided their written informed consent to participate in this study.

## Author Contributions

CPT was in charge of the project, contributed to the conception and design of the work, arranged the learning modules, collected, analyzed, and interpreted the data for the work, and drafted and revised the manuscript. ELJ and ST contributed to conception and design of the project, participant lectures, drafted and revised the manuscript, and supervised the study. CS contributed to the conception and design of the work, held participant lecture, interpreted the data for the work, and drafted and revised the manuscript. CBD, EH, and FM contributed to the conception and design of the work, participant lectures, and drafted and revised the manuscript. All authors contributed to the article and approved the submitted version.

## Conflict of Interest

The authors declare that the research was conducted in the absence of any commercial or financial relationships that could be construed as a potential conflict of interest.
